# Alopecia areata in solid organ transplant recipients: A retrospective analysis

**DOI:** 10.1016/j.jdin.2025.01.014

**Published:** 2025-03-05

**Authors:** Isabella Roman, Miguel A. Aristizabal, Napat Leeaphorn, Leila M. Tolaymat, Thais P. Pincelli

**Affiliations:** a CUNY School of Medicine, New York, New York; b Department of Dermatology, Mayo Clinic Florida, Jacksonville, Florida; c Department of Transplant Nephrology, Mayo Clinic Florida, Jacksonville, Florida

**Keywords:** alopecia, alopecia areata, alopecia totalis, alopecia universalis, epidemiology, immunosuppression, organ transplant, solid organ transplant, solid organ transplant recipients, tacrolimus

*To the Editor:* Alopecia areata (AA) is a chronic, nonscarring autoimmune alopecia with a polygenic basis. Immunosuppression is central to AA treatment, making its occurrence in solid organ transplant recipients (SOTRs), who receive T-cell-inhibiting regimens, rare.^1^ Since 1994, fewer than 20 cases have been reported, most commonly kidney, pancreas, and liver transplants.^2^ AA may have a greater impact on SOTRs’ quality of life due to existing comorbidities.^3^ This study characterizes its prevalence and clinical features at a large academic center.

Following institutional review board approval (IRB# 24-005297), a retrospective analysis of the Mayo Clinic Health System database (October 2005-June 2024) was conducted. SOTRs with a dermatologist-diagnosed AA, with histopathological confirmation in some cases, were included if they had been SOTRs for a year or more. Data on demographics, clinical characteristics, immunosuppressive regimens, AA treatments, and outcomes were collected.

Among 28,260 SOTRs, 21 met inclusion criteria, yielding an AA prevalence of 0.074%. The median age was 48 years (range, 4-75), and 13 (61.9%) were female. The average time from transplantation to AA presentation was 5.25 years, ranging from days to years. Fourteen patients presented with focal AA, 5 with diffuse scalp-hair thinning, and 2 with AA universalis. The scalp was the most common site (Table I). Thoracic transplant recipients had a higher rate of diffuse AA (3, 14.2%) compared to abdominal transplants (1, 4.7%). Treatment options and outcomes varied (Supplementary Table I, available via Mendeley at https://data.mendeley.com/datasets/rn3htwbvh6/1). Autoimmune conditions, including type 1 diabetes, vitiligo, Goodpasture syndrome, and IgA glomerulonephritis, were prevalent in this cohort, consistent with prior reports,^2^ suggesting a potential autoimmune predisposition.

**Table I:** Patient demographics and clinical characteristics

Patient #	Age (y)	Gender	Organ transplant	Reason for transplant	Immunosuppressive regimen	AA timing	AA pattern
1	40	M	K	Type 1 diabetes	Tacrolimus, azathioprine, prednisone	9 y	Alopecia involving the scalp, eyebrows, and genital hair, confirming alopecia universalis.
2	52	F	K	ADPKD	Mycophenolate mofetil, tacrolimus, prednisone, thymoglobulin, basiliximab, alemtuzumab	1 y	Alopecic patches on the scalp.
3	29	M	L + K	Primary hyperoxaluria with bilateral native nephrectomy	Mycophenolate mofetil, tacrolimus, thymoglobulin	6 y	Alopecic patches on the scalp and beard.
4	63	F	K	Polycystic kidney disease.	Mycophenolate mofetil, tacrolimus, prednisone, thymoglobulin	5 y	Alopecic patches on the right vertex and occipital scalp.
5	65	F	K	Tubular sclerosis	Tacrolimus, mycophenolate sodium, thymoglobulin, basiliximab, alemtuzumab	1 y	Alopecic patch on the scalp.
6	25	F	H	Cardiomyopathy	Cyclosporine, azathioprine	9 y	Alopecia involving the scalp, eyebrows, and genital hair, confirming alopecia universalis.
7	69	F	K	ADPKD	Mycophenolate mofetil, tacrolimus, alemtuzumab	7 y	Alopecic patches on the posterior, superior, and right postauricular scalp.
8	34	F	P	Type 1 diabetes	Tacrolimus, prednisone, thymoglobulin, azathioprine, basiliximab	3 y	Alopecic patch on the vertex scalp.
9	58	F	K	IgA nephropathy	Tacrolimus, mycophenolate mofetil, alemtuzumab, thymoglobulin	13 y	Alopecic patches on the left temporal and mid-parietal scalp.
10	8	F	K	Dysplastic kidneys	Tacrolimus, prednisone, thymoglobulin	2 y	Alopecic patches over the scalp.
11	24	M	H	Genetic cardiomyopathy, arrhythmogenic right ventricular dysplasia	Prednisone, sirolimus, thymoglobulin, mycophenolate mofetil	8 y	Diffuse AA involving the entire scalp.
12	16	M	H	Fontan physiology failure	Mycophenolate mofetil, sirolimus	2 mo	Alopecic patch on the scalp.
13	75	F	L + K	NASH, diabetes mellitus-Type II	Tacrolimus, prednisone, azathioprine, basiliximab	5 y	Alopecic patch over the occipital scalp.
14	55	F	L	NASH	Tacrolimus, mycophenolate sodium, basiliximab	3 y	Alopecic patch over the fontal scalp.
15	60	F	H	Nonischemic cardiomyopathy	Tacrolimus, mycophenolate mofetil, sirolimus, basiliximab, thymoglobulin	6 mo	Diffuse AA involving the vertex, frontal, and temporal scalp with preservation of hair on the occipital scalp.
16	52	M	L	AATD	Tacrolimus, mycophenolate mofetil, thymoglobulin	2 y	Alopecic patches on the left anterior hairline; thinning of the right lateral eyebrow.
17	46	F	K + P	Type 1 diabetes	Tacrolimus, prednisone, thymoglobulin	13 y	Diffuse AA with hair thinning involving the entire scalp.
18	8	M	H	Dilated cardiomyopathy	Mycophenolate mofetil, prednisone, tacrolimus, thymoglobulin	29 d	Diffuse AA over the scalp with a focal alopecic patch on the left posterior parietal scalp.
19	4	M	K	Posterior urethral valves	Mycophenolate mofetil, tacrolimus, thymoglobulin	2 y	Alopecic patch on the right superior occipital scalp.
20	48	M	K + P	Type 1 diabetes	Tacrolimus, mycophenolate sodium, prednisone	20 y	Alopecic patch over the left cutaneous upper lip.
21	58	F	K + H	Cardiorenal syndrome, ischemic cardiomyopathy	Tacrolimus, mycophenolate sodium, prednisone, basiliximab	7 mo	Diffuse AA involving the entire scalp

AA timing: Time from transplant to the presentation of alopecia areata.

*AA*, Alopecia areata; *AATD*, alpha-1 antitrypsin deficiency; *ADPKD*, autosomal dominant polycystic kidney disease; *F*, female; *H*, heart; *K*, kidney; *L*, liver; *M*, male; *NASH*, nonalcoholic steatohepatitis; *P*, pancreas.

The role of systemic immunosuppression in AA development remains uncertain. Notably, 18 (85%) patients were on tacrolimus, which has been linked to drug-induced alopecia and AA.^4^ None of our patients received cyclosporine, although prior studies have suggested a relationship between declining cyclosporine levels and AA.^1^ Patients were treated with 2 or 3 immunosuppressants, which theoretically may control AA, but no medication patterns were clearly associated with its development. Unlike previous research,^2^ we report on transplant induction therapies, which suppress T-cell activity for extended periods posttransplantation. Lymphodepletion from induction therapy may lead to homeostatic activation of new T cells, potentially causing autoimmunity, as seen with conditions like Graves’ disease.^4^

Another notable finding was the late onset of AA after transplantation in most patients. This may reflect the initial use of high-dose immunosuppressants, which are tapered over time and may have suppressed AA development. Interestingly, the prevalence of AA in our SOTR cohort (0.074%) was lower than in the general US population (0.1% to 0.2%).^5^ This discrepancy may reflect the ability of immunosuppressive regimens to initially mitigate the autoimmune response associated with AA.

The exact pathogenic mechanism of AA in SOTRs remains unclear, likely influenced by the complex immunologic landscape of these patients. Limitations of this study include the small sample size, single-center data, and retrospective design. Additionally, the prevalence of AA may be underestimated due to reliance on database diagnoses and lack of biopsy confirmation in all cases. Further research is needed to better understand AA in SOTRs to improve patient care.

## Conflicts of interest

None disclosed.

## References

[1] Phillips M., Graves J., Nunley J. (2005). Alopecia areata presenting in 2 kidney-pancreas transplant recipients taking cyclosporine. J Am Acad Dermatol.

[2] González-Guerra E., Taboada A.C., Muñoz L.C., Fructuos A.I.S., Bran E.L. (2019). Does immunosuppression help control alopecia areata? A study in transplant recipients. Skinmed.

[3] Tarabeih M., Bokek-Cohen Y., Azuri P. (2020). Health-related quality of life of transplant recipients: a comparison between lung, kidney, heart, and liver recipients. Qual Life Res.

[4] Zuk D.M., Koh A., Imes S., Shapiro A.M., Senior P.A. (2011). Three cases of alopecia following clinical islet transplantation. Am J Transplant.

[5] Benigno M., Anastassopoulos K.P., Mostaghimi A. (2020). A large cross-sectional survey study of the prevalence of alopecia areata in the United States. Clin Cosmet Investig Dermatol.

